# Obituary: Dr. Eugene Garfield (1925-2017)

**DOI:** 10.11604/pamj.2017.26.160.12124

**Published:** 2017-03-20

**Authors:** Allan Mwesiga

**Affiliations:** 1 The Pan African Medical Journal

**Keywords:** Obituary, citation analysis, journal impact factor, citation index

## Obituary

**Figure:**
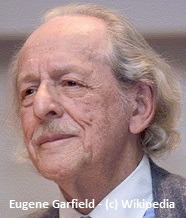


It is with great sadness that we announce the death of Dr. Eugene Garfield. Dr. Garfield died on Sunday 26 February 2017. He was 91. Dr. Garfield is credited as the inventor of the journal impact factor [1]. He founded the Institute for Scientific Information (ISI) in 1955 [1, 2]. The ISI’s Scientific Citation Index (SCI),which was made available electronically as the Web of Science, and other citation index databases have helped link the body of scientific literature and provide an efficient means for scientists to look up search existing scientific literature [1, 2]. In 1982, the Thomson Reuters Corporation acquired the SCI [1]. Clarivate Analytics has owned the division since 2016 [3]. Dr. Garfield also founded two publications “Current Contents” and “The Scientist” to compile news and information for scientists and researchers [1, 2]. Dr. Garfield was awarded a PhD in structural linguistics from the University of Pennsylvania in 1961 [1]. He is survived by his wife, Meher, three sons, a daughter, a step-daughter, two granddaughters, and two great-grandchildren [1].
